# Towards Virtual VATS, Face, and Construct Evaluation for Peg Transfer Training of Box, VR, AR, and MR Trainer

**DOI:** 10.1155/2019/6813719

**Published:** 2019-01-06

**Authors:** Zhibao Qin, Yonghang Tai, Chengqi Xia, Jun Peng, Xiaoqiao Huang, Zaiqing Chen, Qiong Li, Junsheng Shi

**Affiliations:** ^1^Yunnan Key Laboratory of Opto-Electronic Information Technology, Yunnan Normal University, Kunming 650000, China; ^2^Department of Thoracic Surgery, Yunnan First People's Hospital, Kunming 650000, China

## Abstract

The aim of this study is to develop and assess the peg transfer training module face, content and construct validation use of the box, virtual reality (VR), cognitive virtual reality (CVR), augmented reality (AR), and mixed reality (MR) trainer, thereby to compare advantages and disadvantages of these simulators. Training system (VatsSim-XR) design includes customized haptic-enabled thoracoscopic instruments, virtual reality helmet set, endoscope kit with navigation, and the patient-specific corresponding training environment. A cohort of 32 trainees comprising 24 novices and 8 experts underwent the real and virtual simulators that were conducted in the department of thoracic surgery of Yunnan First People's Hospital. Both subjective and objective evaluations have been developed to explore the visual and haptic potential promotions in peg transfer education. Experiments and evaluation results conducted by both professional and novice thoracic surgeons show that the surgery skills from experts are better than novices overall, AR trainer is able to provide a more balanced training environments on visuohaptic fidelity and accuracy, box trainer and MR trainer demonstrated the best realism 3D perception and surgical immersive performance, respectively, and CVR trainer shows a better clinic effect that the traditional VR trainer. Combining these in a systematic approach, tuned with specific fidelity requirements, medical simulation systems would be able to provide a more immersive and effective training environment.

## 1. Introduction

Video-assisted thoracoscopic surgery (VATS), which is the most common minimal invasive surgery (MIS) therapy for lung carcinomas [1–3], is the most widespread cancer in the world with only approximately 16% five-year survival rate. Furthermore, hospitalization outcomes show the patients' quality of life (QoL) and follow-up adjuvant chemotherapy endurance that are significantly promoted compared with traditional thoracotomy, without interfering with survival outcomes [4, 5]. Peg transfer training, as one of the essential modules of the fundamental thoracoscopic surgery curriculum, is the compulsory test requirement before the surgeries take the American Board of Surgery examination [6, 7]. With two Maryland clamps, trainees need to pick up six tiny blocks with nondominate hand, transfer to the other hand, and place them stably on the other side peg, respectively, whereby to achieve the bimanual dexterity and eye-hand coordinative skills training [7, 8]. The surgical simulators can be divided into box trainer, VR trainer, AR trainer, and MR trainer, and each class has advantages and disadvantages. The Box trainer is the traditional surgical training framework based on a real physical model that is portable and easy to operate; however, the disadvantage is this kind of trainer cannot be reused for multitimes. VR-based surgical simulations have attracted many researchers' attentions over the years and gradually turned into a real-life medical training simulation solution, providing repeatable training experience, without ethical or hygienic issues [9–11], and the disadvantage of this simulator is lack of high immersive visual and haptic rendering algorithms. Due to the recent advances in the field of AR and MR, cognitive sense has brought into the next level of surgical simulator with enhanced immersion and interactivity [12–14]; however, there are also several defects of these simulators, such as the high price, visual uncomfortable, and lack of the real surgical environment fidelity. Maciel et al. developed a virtual reality laparoscopic skill trainer named VBLaST, with real-time evaluation function for the peg transfer [15], and the Lap Mentor™ simulator [16, 17]. Loukas et al. compared the AR-based peg transfer with the box simulator and VR simulator [6], and Huber et al. added a highly immersive 360° real operating room environment to construct an MR training environments [18, 19]. Nevertheless, there is no comparative study that has focused box, AR, VR, CVR, and MR simulators on the peg transfer training, in another word, which one is the most effective simulator to shorten the surgical learning curve [20, 21]. The aim of this research is to determine which simulator is better by the advantages and disadvantages of the five simulators that were compared through the evaluation results of the face and content and construct. There are three main innovative contributions in this study, and the overarching one is we compared the experimental data and simulation results among five different kinds of trainers of peg transfer training and summarized the conclusion which may benefit for the advanced research of the virtual surgery. The second one is we addressed a full immersive and accurate scenario to peg transfer training with a detailed assessment tool, and the last one is we addressed the detailed design of the first commercial available VATS VR simulator in both hardware and the training implementation.

The structure and content of this article are organized as follows: we briefly make an introduction on peg transfer training and challenges of virtual medical training in the abovementioned part, after that, we review the previous related works on the virtual peg transfer simulator. Thirdly, the simulator design, with virtual training evaluation experiments, is designed in the methodology part. Fourthly, the experimental data and evaluation results are demonstrated and discussed. Finally, we summarize the results and the potential contributions this paper makes.

## 2. Materials and Methods

### 2.1. Simulator Design

#### 2.1.1. Hardware

**Figure 1:**
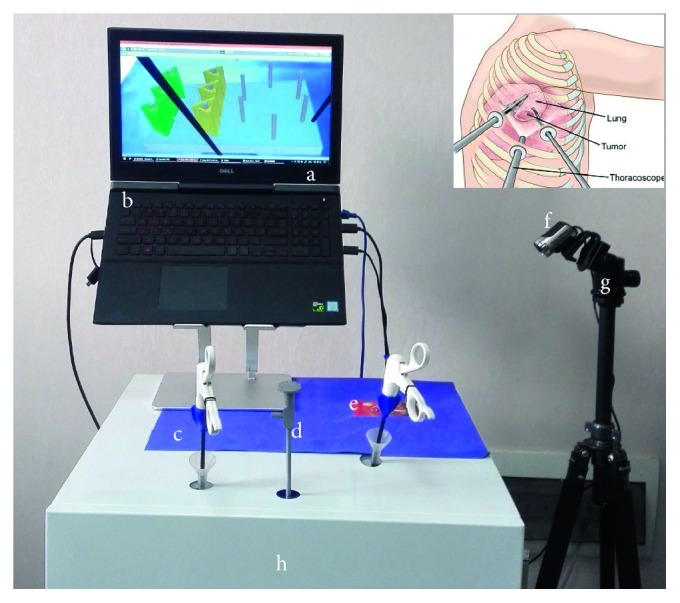
The immersive training platform for virtual peg transfer consists of (a) guide video, (b) a laptop with i7 6700 (3.4 GHz) CPU, 4 GB memory, and 1070 NVIDIA graphics GPU, (c) surgical clamp, (d) endoscope, (e) AR tracking target, (f) AR camera, (g) camera tripod, and (h) haptic devices (PHANTOM Omni).

In the light of the development of commercial VR-based laparoscopic surgical simulators, LapSim® (Surgical Science, Sweden) and LAP Mentor (3D System, USA) [1, 15], and after observed considerable VATS lobectomy operations in the thoracic surgery department of Yunnan First People's Hospital, we chose the three-port standardized anterior VATS surgery as the simulated content. As the commonest surgical approach in lung carcinomas lobectomy, the three-port VATS are described as opened 3 single diameters of 1.5 cm incisions in the side of the patient's thorax: one is for endoscopic camera recording the operation field and the other two are the operative tunnels for surgical instruments stretching in and out to dissect and staple the lesion [2, 16]. The surgical simulator is called VatsSim-XR, as shown in Figure 1, based on the procedures need to be simulated in the abovementioned, and hardware of VatsSim-XR (VR, AR, and MR) is 60 × 67 × 160 cm and principally composed of a 24-inch naked eye 3D display, a footswitch pedal, two VATS surgical devices connected with dual haptic devices with one held as the drag instrument and the another mimics the stapling device, an endoscope connected with a 3D mouse (3Dconnexion, Germany), an HMD, and a workstation. To improve the surgical immersion, we proposed a solution to incorporate multiple higher-fidelity factors towards a surgeon's sensations (vision, touch, and hearing) during practical surgery to achieve total immersion. We developed a versatile simulation platform VatsSim-XR that is able to implement 5 different simulation modes, specifically, the AR and MR modes. To ensure the same training block between the real and virtual peg transfer platform, the .STL file is exported from the blender firstly, and then, the 3D printer is employed to print all the training pegs for the box training simulator. For the AR peg transfer simulation, we utilized a web camera Logitech CC2900ep HD1080p as the detected sensor for the display, and for the CVR training and the MR training, HTC VIVE (HTC Corporation, Taiwan, China) is the first virtual reality helmet, and it is used to show the surgical environments [22]. Especially, for the real surgical environments rendering, we employed the Samsung Gear 360 (Samsung, South Korea) to record the 360° video in the operation room (OR) of an upper-right lobe VATS in Yunnan First People's Hospital [18, 19, 23, 24].

#### 2.1.2. Software

**Figure 2:**
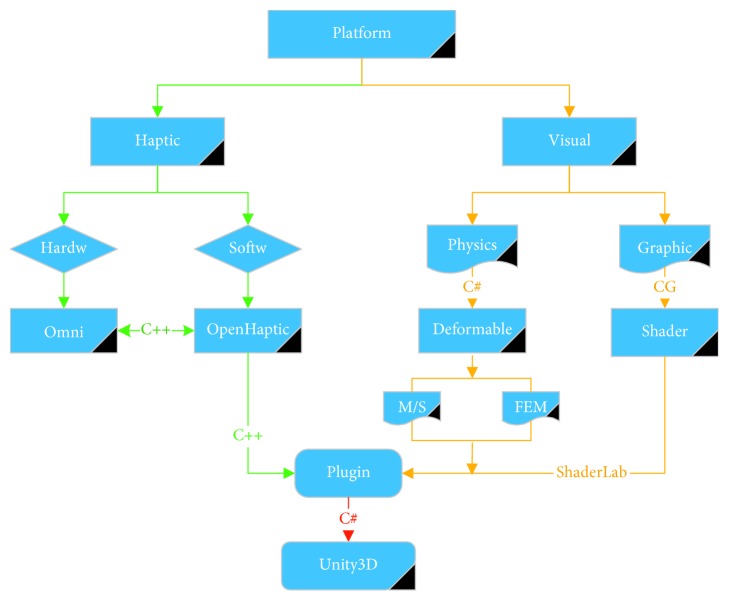
Visual and haptic rendering pipelines of the virtual peg transfer simulator.

We designed a framework for the implementation of peg transfer training simulation, as shown in Figure 2. The virtual simulator mainly includes two parts: visual (physics and graphic) and haptic (PHANTOM Omni hardware and OpenHaptic software). The corresponding OpenHaptic function will be invoked when pressing the physical button of PHANTOM Omni (Geomagic, USA) or there is contact between the virtual surgical instrument and the model with a rigid body, and then, there will have a corresponding force feedback. There are physics with rigid or deform and graphics with a shader in the visual aspect. We combine the visual plugin, such as Bullet, AR kit and SteamVR, and the haptic plugin OpenHaptic into the Unity3D (version 2017.3.1) for simulation training.

### 2.2. Participants

The evaluation cohort of 32 trainees comprising 24 novices and 8 experts underwent the peg transfer procedures on the five simulators conducted in the department of thoracic surgery of Yunnan First People's Hospital. Both expert and novice trainers firstly receive a didactic teaching from a developer of these simulators with the tasks and techniques of peg transfer. The medical experience of the novice group is 3 to 6 years and the expert group is 11 to 30 years. To evaluate if the virtual game experience may affect the training result, the experience of using both VR and HMD had been recored during the experiments. The demographic details of the trainees are shown in Table 1.

### 2.3. Simulator Tasks

**Figure 3:**
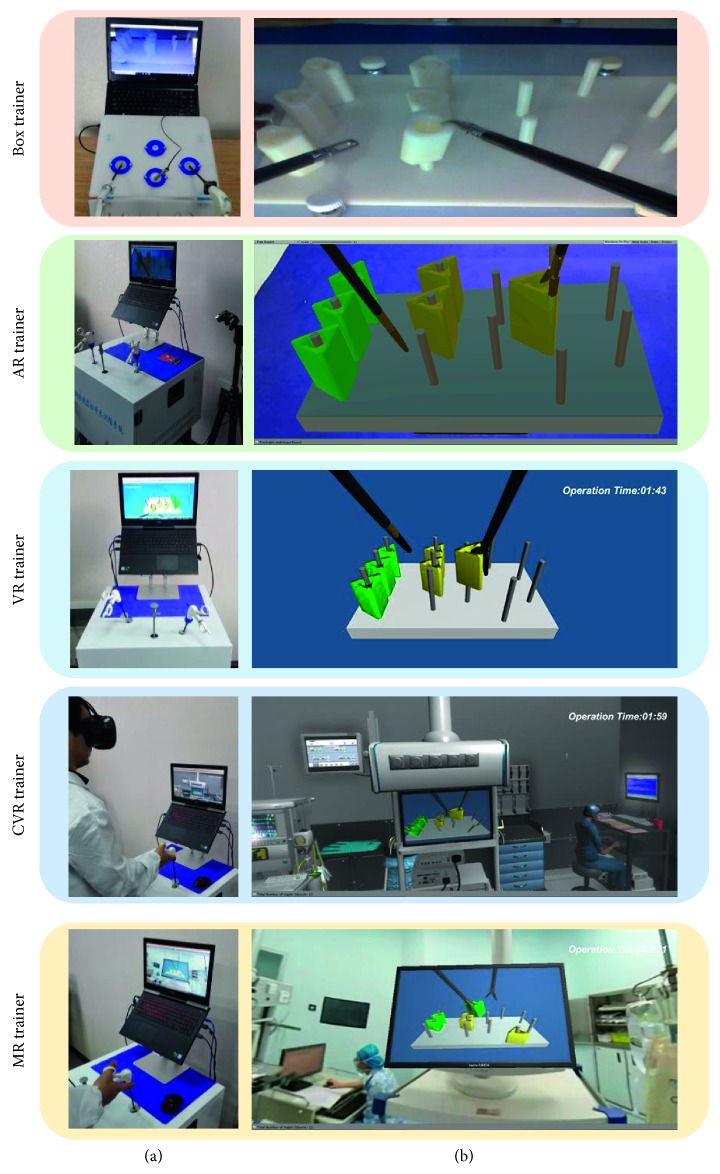
Peg transfer training platform. (a) The manipulative platform. (b) The operation interface. CVR training and MR training need the HMD device, and the AR training needs the camera module.

Both novice and expert groups after didactic training session are towards to evaluate the simulator validity. The order of training is the box, VR, CVR, AR, and MR, and the 30-minute break should be interspersed between each peg transfer simulator test. Firstly, the trainee adjusted the virtual endoscope to reach the proper position and angle to obtain the best viewing perspective of the operating scene; after that, two clamps are used to grasp and transfer the block model on the peg base. The left-hand clamp grabs the block on the left side and passes it to the right clamp; then, the right clamp places the block on the right side, which is demonstrated in Figure 3.

**Figure 4:**
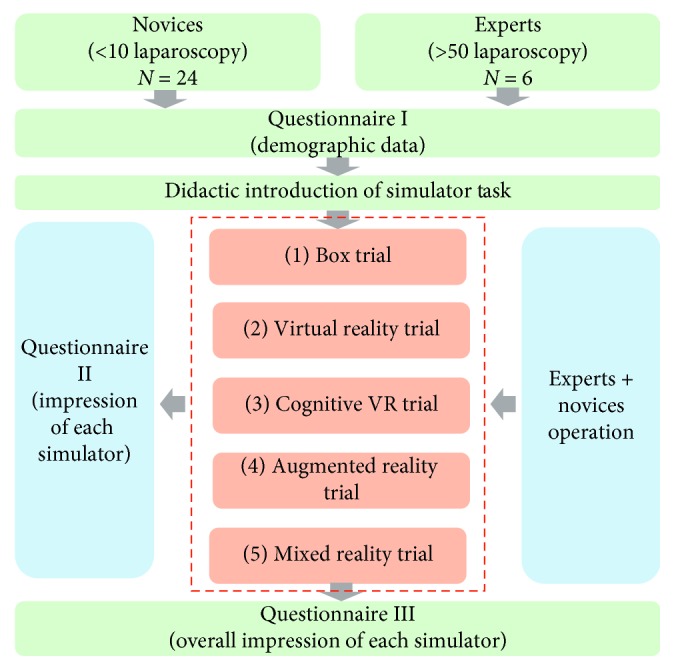
Evaluation system procedures design underwent our immersive virtual peg transfer simulator.

After the aforementioned experiments, all 32 trainees' performance is recorded by the objective questionnaires to evaluate the detailed surgical skills and be compared with each other. Subjective questionnaires also need to be filled to evaluate the face and content validity of five simulators, and the detailed evaluation flow chart is demonstrated in Figure 4.

### 2.4. Evaluation

A questionnaire consisting of 13 questions about the visual and haptic aspects of the simulators was created for face and content assessment validity. Due to the lack of experience of the novice, subjective judgment may have a large error in the understanding of the simulator. Face and content validities were established by 8 experts that standardized subjective questionnaires parameters. This questionnaire utilizes 5-point Likert-type scales to evaluate the visual and haptic of the simulators (1 = poor to 5 = excellent). The detail subjective questionnaire of the face and content validity is demonstrated in Table 2. The objective evaluation of the construct has six assessed items that are totally operation time (T), surgical clamps track length (CL), endoscope track length (EL), surgical clamps angle accumulation (CA), endoscope angle accumulation (EA), and the numbers of block drop (ND). First, verify whether each set of data obeys a normal distribution, and then compare the performance of multiple simulators under six parameters.

### 2.5. Data Analysis

In terms of face and content validity, descriptive statistics were used to analyze the questionnaire data by calculating the mean value of subjective question and their standard errors. For the construct validity, assessing the test results of novice and expert on six parameters of five simulators with Shapiro–Wilk test, *p* > 0.05, is considered to be subject to normal distribution. Box plots are used for analysis of objective parameters. Each group of T of novices and experts on the five simulators follows a normal distribution and was compared between groups using an independent-sample *t*-test, and each group of T of five simulators on novices and experts was compared between groups using the one-way ANOVA test. But, most of the CL, EL, CA, EA, and ND were not a normal distribution, each group of those data of novices and experts on the five simulators was compared between groups using the two-tailed Mann–Whitney *U* test, and each group of those data of five simulators on novices and experts was compared between groups using the Kruskal–Wallis test. A *p* value < 0.05 was considered significant. Analyses were performed using the software package, IBM SPSS Statistics, version 20.0.

## 3. Results

### 3.1. Face and Content Validation

**Figure 5:**
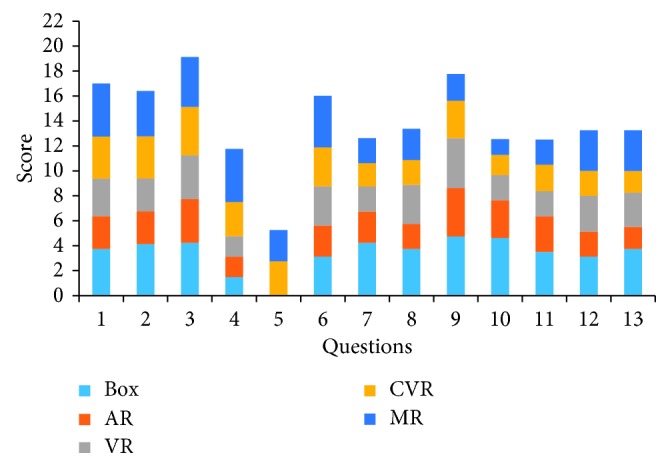
Face and content validity score of the expert group on these five simulators.

Aiming to the perceptions of visual and tactile sensations during the use of the five simulators, we set up a subjective questionnaire with 13 questions using the scoring method of the 5-point Likert-type scale. After the experiment of the simulation operation, the questionnaire was filled by 8 experts for the experience during the operation. Through the Shapiro–Wilk test, each set of score data is an approximately normal distribution. The thirteen subjective questionnaires were collated, and the score data of each simulator were assessed by the Shapiro–Wilk test. The average and standard deviation of each set of data are shown in Table 3, and the distribution of the average score is shown in Figure 5. It can be found that the box and MR scores demonstrated a higher score than the AR and VR scores in a visual sense. In terms of haptic sense, the box scores are higher than the AR, VR, CVR, and MR scores, which demonstrated the best haptic immersion among these virtual simulators.

### 3.2. Construct Validation

**Figure 6:**
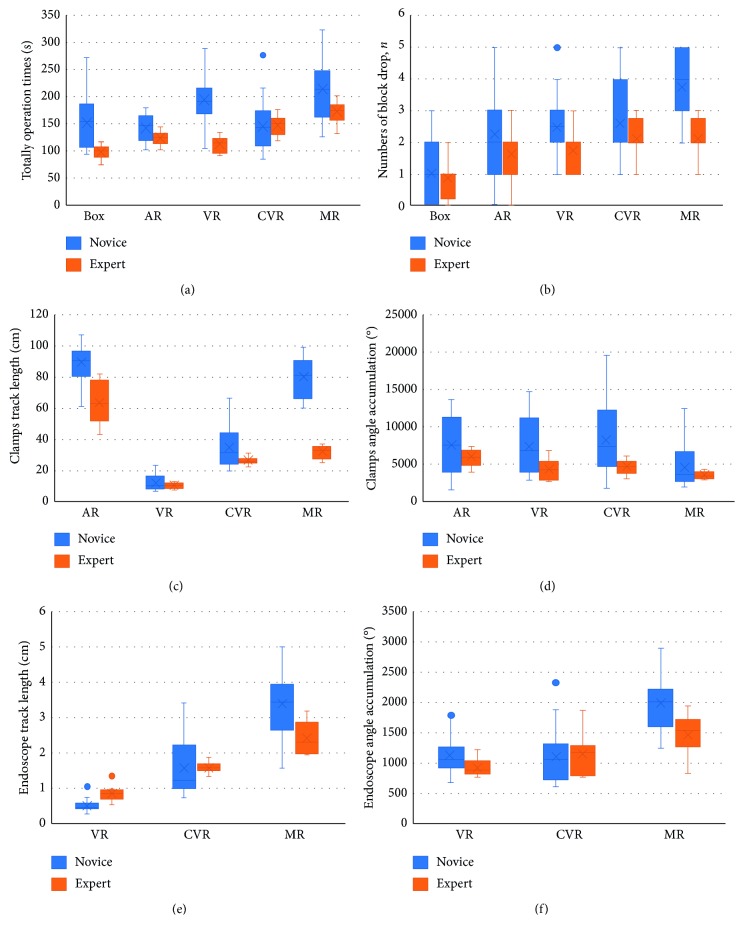
Performance comparison of six parameters in five simulators. (a) T: totally operation time. (b) ND: numbers of block drop. (c) CL: surgical clamps track length. (d) CA: surgical clamps angle accumulation. (e) EL: endoscope track length. (f) EA: endoscope angle accumulation.

**Figure 7:**
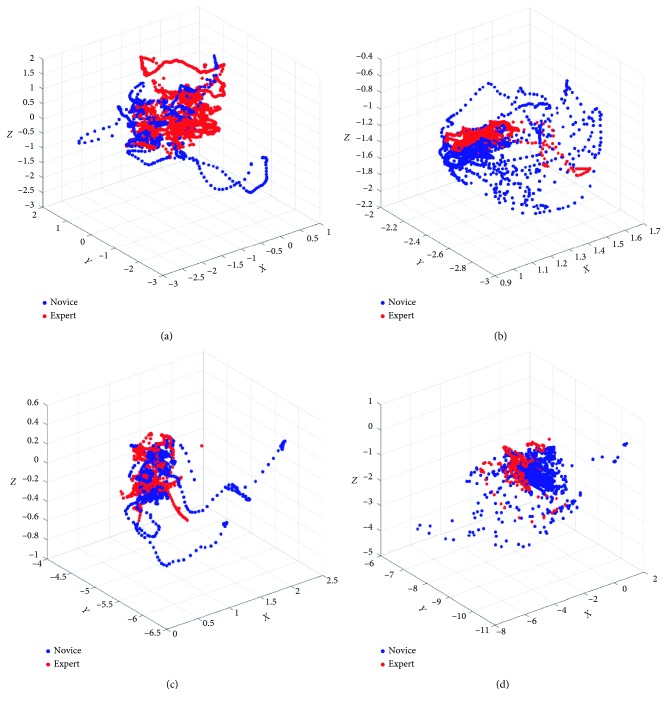
Clamp moving trajectory comparison during the simulation. (a) VR group. (b) AR group. (c) CVR group. (d) MR group.

During the operation of 32 trainees, the VR, CVR, AR, and MR simulators automatically recorded the trajectory length and rotation angle of the surgical instruments and endoscopes in real time, as well as the total time spent and the numbers of block drop on each operation. However, the box simulator cannot record the length and angle of the motion track and the numbers of the block drop, and the total time of the operation can only be roughly recorded by the timer. Six box plots for the experimental data of six parameters T, CL, EL, CA, EA, and ND were made by SPSS, and those box plots which include mediums and means are shown in Figure 6. Similarly, after calculating these *p* values of the parameters of the different simulators for the novice and expert groups, the results are shown in Table 4. Most of the evaluation parameters of the expert group are lower than that of the novice group in each simulator. *p* < 0.05, which is only for the EL of VR simulator. EL and EA of CVR simulator data of the expert group demonstrated a higher score than that of the novice. Comparing VR, CVR, and MR scores of the expert group, those scores of the VR group demonstrate the best performance. The clamp moving trajectory of AR, VR, CVR, and MR groups during the simulation is demonstrated in Figure 7, and it shows that the constructive difference between these four training groups and the AR group demonstrated a better-concentrated trajectory than other groups.

## 4. Discussion

There is an increasing use of simulators to learn, improve, and rehearse surgical skills in medical training. The VatsSim-XR is a versatile simulator that is able to perform multiple surgical training scenarios and implement 4 different simulation modes (VR, CVR, AR, and MR) on a single device. The Society of American Gastrointestinal and Endoscopic Surgeons (SAGES) and the American College of Surgeons (ACS) have established a standard for fundamentals of laparoscopic surgery (FLS) training and surgical skills assessment [25–27]. The FLS consists of five basic foundations: peg transfer, pattern cutting, ligation loop, and suturing with either intracorporal or extracorporeal knot tying, and these tasks are designed to train the operational skills and assessment of medical interns and residents. Peg transfer is the basic training task to train the dual-hands coordination and the hand-eye coordination for the medical interns and residents [8]. FLS simulation training is mostly based on box and VR simulators in the market.

In terms of haptic, the box simulator is superior to the other four simulators, but an operation object is an object that is easily destroyed after repeated use. In terms of visual, the box and MR simulators are better. The AR simulator is the best in terms of interactive, and in the interactive environment part, where the simulator is more immersive: MR simulator > CVR simulator; however, the box, VR, and AR simulators have almost no surroundings and are less immersive.

Compared with novice groups of six parameters on five simulators, most of the expert groups show short operation time, short track length, small angle accumulation, and small drop number, but only these three groups, the EL of the VR simulator, EL, and EA of CVR the simulator, show the opposite result, and the main reason is that when adjusting the endoscope, the novice does not move and rotate the endoscope to an optimal viewing angle in strict accordance with the surgical standard, resulting in greater difference. For the VR, CVR, and MR simulators of six parameters, the data size of expert groups basically follows a rule VR < CVR < MR, but only the MR simulator data of the CA parameter are smaller than VR and MR simulators, because the experts have adapted to the 360° real operating room scene in the MR simulator in advance. Although the MR simulator is not perfect in terms of T, CL, EL, CA, EA, and ND, it provides a highly realistic operating room for hospital interns and residents. The environment, closer to the real surgery site, has great potential for development. Experiments and evaluation results conducted by both professional and novice thoracic surgeons show that the AR trainer is able to provide a more balanced training environment on visuohaptic fidelity and accuracy, the box trainer and MR trainer demonstrated the best realism 3D perception and surgical immersive performance, respectively, and the CVR trainer shows a better clinic effect that the traditional VR trainer.

The advantages and disadvantages of the five simulators are shown in Table 5. The haptic, visual, and surrounding environments are compared separately by levels (III means the highest and I means the lowest). The complex design of our integrated system will provide a more immense and effective training environment for the medical surgery simulation.

## 5. Conclusions

In this paper, we have detailed a virtual surgical educative simulator with realistic performance in both visual and haptic sensations for the peg transfer procedures, build the face, content and construct validation on the peg transfer training by use of the box, VR, AR, and MR trainer, thereby to compare advantages and disadvantages of these simulators. However, during the interaction, the sense of touch is not immersive enough; furthermore, there is a cross between the gripper and the interacting virtual object. What is more, in the comparison experiments design, the box simulator is not able to automatically record the trajectory and rotation angle parameters of the instrument and endoscope, which means the comparison of the six parameters is not comprehensive enough in this manuscript. In the future works, we will improve the visual and haptic experiment design parts to achieve a high immersive and realistic simulation environment both visual and haptic perception.

## Figures and Tables

**Table 1:** Demographic data of the thoracic surgery trainees.

	Group A (novices)	Group B (experts)
Number	24	8
Age (years)	25.5 (24–29)	42.2 (39–56)
Postgraduate year of training	4 (3–8)	12 (11–30)
Male (%)	92	83
Right-handed (%)	100	87
Box trainer experienced	<10	>50
VR game experienced	5/24	1/8
HMD experienced	3/24	1/8

**Table 2:** Subjective questionnaire of the face and content validity.

Face and content validity questions (score: 1–5, 1 = poor to 5 = excellent)
Q1: Realism of peg model (visual)
Q2: Realism of endoscope model (visual)
Q3: Realism of surgical clamps (visual)
Q4: Realism of surgical environment (visual)
Q5: Comfortable of training content in HMD (visual)
Q6: Overall realism of visualization
Q7: Realism of peg manipulation (haptic)
Q8: Realism of endoscope manipulation (haptic)
Q9: Realism of surgical clamps manipulation (haptic)
Q10: Realism of interaction between the surgical clamps and peg (haptic)
Q11: Overall realism of manipulation
Q12: I would like to recommend this simulator to VATS surgical training for medical students
Q13: I would like to recommend this simulator as an assessment tool for VATS surgical skills

**Table 3:** Subjective questionnaire results of the face and content validity (experts group).

Questionnaires	Box		AR		VR		CVR		MR	
	Mean	SD	Mean	SD	Mean	SD	Mean	SD	Mean	SD
	Score (1–5)									
Q1	3.75	0.71	2.63	0.92	3.00	0.53	3.38	0.92	4.25	0.71
Q2	4.13	0.64	2.63	0.92	2.63	0.91	3.38	0.92	3.63	0.92
Q3	4.25	0.71	3.50	0.93	3.50	0.53	3.88	0.64	4.00	0.76
Q4	1.50	0.76	1.63	0.74	1.63	0.74	2.75	0.71	4.25	0.71
Q5	—		—		—		2.75	1.04	2.50	0.93
Q6	3.13	0.64	2.50	0.93	3.13	0.64	3.13	0.64	4.13	0.64
Q7	4.25	0.89	2.50	0.93	2.00	0.75	1.88	0.64	2.00	0.76
Q8	3.75	0.71	2.00	0.76	3.13	0.83	2.00	0.76	2.50	0.93
Q9	4.75	0.46	3.88	0.64	4.00	0.76	3.00	0.76	2.13	0.64
Q10	4.63	0.52	3.03	0.64	2.00	0.76	1.63	0.74	1.25	0.46
Q11	3.50	0.93	2.88	0.64	2.00	0.76	2.13	0.64	2.00	0.76
Q12	3.13	0.64	2.00	0.76	2.88	0.64	2.00	0.76	3.25	1.04
Q13	3.75	0.71	1.75	0.71	2.75	1.04	1.75	0.71	3.25	0.89

**Table 4:** The comparison of objective parameters for construct validity (experts and novices).

		*p* value					
		T	CL	EL	CA	EA	ND
Box	Experts	0.002	—	—	—	—	0.781
	Novices						

AR	Experts	0.053	0.000	—	0.277	—	0.302
	Novices						

VR	Experts	0.000	0.761	0.001	0.024	0.037	0.084
	Novices						

CVR	Experts	0.744	0.041	0.177	0.030	0.486	0.405
	Novices						

MR	Experts	0.045	0.000	0.003	0.965	0.009	0.001
	Novices						

*p* value	Experts	0.000	0.000	0.000	0.005	0.012	0.014
	Novices	0.000	0.000	0.000	0.013	0.000	0.000

**Table 5:** Comparisons of the five peg transfer simulators.

	Advantages	Disadvantages	Immersive		
			Haptic	Visual	Environments
Box	Realistic force feedback Realistic 3D perception Cost-effective Portable and easy to use	Low integration Lack of objective assessment Low immersion Without OR environment	III	I	I

VR	Objective assessment High interactivity Unlimited use	Low immersion Without OR environment Low 3D perception	I	I	I

CVR	Objective assessment High interactivity High immersion Motion track (HMD)	Virtual OR environment Narrow FOV Low 3D perception	I	II	II

AR	Objective assessment High interactivity High immersion Realistic 3D perception	Low immersion Without OR environment Environmental influence	I	I	I

MR	100% OR environment Objective assessment High interactivity High immersion	OR environment distortion Narrow FOV	I	III	III

## Data Availability

The data used to support the findings of this study are available from the corresponding author upon request.

## Supplementary Materials

Supplementary Materials The supplementary video demonstrated the peg transfer experimental procedures on the box, VR, CVR, AR, and MR trainer.
